# A “Vascular Band-Aid” strategy for proactive inhibition of post-anastomotic vascular complications

**DOI:** 10.1093/rb/rbag164

**Published:** 2026-08-04

**Authors:** Ruijie Xu, Jingqiang Yan, Xiaojuan Li, Xuelian Yin, Zhao Yang, Qihui Zhou, Bingcheng Yi

**Affiliations:** Department of Vascular Surgery, Qingdao Municipal Hospital, School of Life Sciences and Health, NHC Key Laboratory of Cardiopulmonary Rehabilitation and Functional Recovery, University of Health and Rehabilitation Sciences, Qingdao 266113, China; Department of Vascular Surgery, Qingdao Municipal Hospital, School of Life Sciences and Health, NHC Key Laboratory of Cardiopulmonary Rehabilitation and Functional Recovery, University of Health and Rehabilitation Sciences, Qingdao 266113, China; Department of Vascular Surgery, Qingdao Municipal Hospital, School of Life Sciences and Health, NHC Key Laboratory of Cardiopulmonary Rehabilitation and Functional Recovery, University of Health and Rehabilitation Sciences, Qingdao 266113, China; School of Pharmacy, Qingdao University, Qingdao 266071, China; Department of Vascular Surgery, Qingdao Municipal Hospital, School of Life Sciences and Health, NHC Key Laboratory of Cardiopulmonary Rehabilitation and Functional Recovery, University of Health and Rehabilitation Sciences, Qingdao 266113, China; School of Pharmacy, Qingdao University, Qingdao 266071, China; Department of Vascular Surgery, Qingdao Municipal Hospital, School of Life Sciences and Health, NHC Key Laboratory of Cardiopulmonary Rehabilitation and Functional Recovery, University of Health and Rehabilitation Sciences, Qingdao 266113, China; Department of Vascular Surgery, Qingdao Municipal Hospital, School of Life Sciences and Health, NHC Key Laboratory of Cardiopulmonary Rehabilitation and Functional Recovery, University of Health and Rehabilitation Sciences, Qingdao 266113, China; School of Pharmacy, Qingdao University, Qingdao 266071, China; Department of Vascular Surgery, Qingdao Municipal Hospital, School of Life Sciences and Health, NHC Key Laboratory of Cardiopulmonary Rehabilitation and Functional Recovery, University of Health and Rehabilitation Sciences, Qingdao 266113, China; School of Pharmacy, Qingdao University, Qingdao 266071, China

**Keywords:** vascular anastomosis, Vascular Band-Aid, inflammation, disordered repair, surface engineering

## Abstract

Vascular anastomosis frequently fails in elderly patients or those with chronic comorbidities due to aberrant inflammation and disordered repair, ultimately leading to stenosis or occlusion. Inspired by the dual action of a conventional band-aid: physical protection and microenvironmental intervention, we herein develop a “Vascular Band-Aid” strategy designed to target the anastomotic site through interface intervention and proactively accelerate incised vessel healing to prevent adverse events. This band-aid, featuring a bioactive interface, is constructed through stepwise modification of aligned poly(L-lactide-co-ε-caprolactone) nanofibrous membrane with copper-chelated tannic acid complexes (*via* noncovalent binding) and poly-L-lysine molecules (*via* Michael-type addition and Schiff-base reactions). The resulting coating markedly increases fiber surface roughness, enhances membrane wettability, surface charge and protein adsorption, owing to the introduction of abundant –NH_2_ and phenolic hydroxyl groups. These features improve cell affinity and drive endothelial mature through cell–matrix interactions and mechanotransduction, while alleviating inflammation-induced cytotoxicity. When applied at anastomotic sites in an arterial intimal hyperplasia model, the band-aid provides durable structural support, modulates inflammation and accelerates cell proliferation to enhance vascular regeneration, effectively demonstrating its efficacy in preventing anastomotic failure. This surface-engineering strategy also establishes a generalizable paradigm for other tissue interfaces where postoperative healing remains a critical barrier.

## Graphical abstract

**Graphical Abstract:**
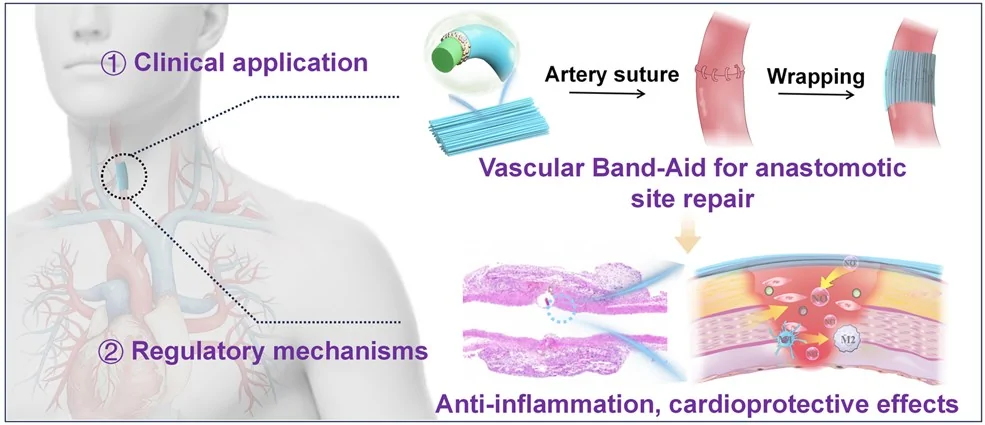

## Introduction

Vascular anastomosis serves as the cornerstone of cardiovascular, reconstructive and transplant surgeries [1], yet it remains the predominant site of postoperative failure. Despite refinements in microsurgical techniques, complications such as acute thrombosis, intimal hyperplasia and pseudoaneurysm formation continue to arise predominantly at the suture sites [2, 3], a vulnerability exacerbated in elderly patients and those with chronic comorbidities. Pathophysiologically, surgical trauma disrupts the local tissue microenvironment, triggering a self-perpetuating cascade characterized by aberrant inflammatory signaling (e.g. involving nuclear factor kappa-B and NOD-like receptor protein 3 pathways [4, 5]), dysregulated proliferation and migration of vascular smooth muscle cells and profound endothelial dysfunction, ultimately culminating in luminal stenosis or occlusion. Current clinical strategies remain largely confined to optimizing suturing techniques or systemic pharmacotherapy, which is a passive, diffuse approach [1, 6]. Neither adequately modulates the pathological microenvironment at the anastomotic interface: refined suturing lacks biological intervention, whereas systemic drugs suffer from off-target toxicity and poor local bioavailability. An ideal, yet largely unrealized, therapeutic paradigm would be a localized, actively responsive intervention that preemptively inhibits thrombosis or intimal hyperplasia before it occurs at the anastomotic sites.

Inspired by the dual function of a conventional band-aid, which physically isolates a wound from external mechanical insults while establishing an optimal environment for immune and host cell crosstalk, we propose a functional “Vascular Band-Aid.” This concept transcends passive physical protection, offering a multimodal, spatiotemporally controlled therapeutic platform for precision targeting of the anastomotic sites. By delivering localized, sustained modulation of the pathological cascade (e.g. simultaneously achieving anticoagulation, immune regulation and accelerated vascular repair), this approach aims to proactively intercept the core drivers of anastomotic failure at their source, thereby breaking the cycle of postoperative vascular dysfunction. Biomaterials offer distinct advantages over conventional pharmacotherapy and surgical techniques [7, 8]. Their intrinsic physicochemical properties can actively modulate host cellular responses and immune microenvironments to accelerate tissue repair, while their surfaces can be rationally engineered through bioactive molecule immobilization or controlled-release coatings to enable precise, spatiotemporally regulated interventions [9, 10]. These attributes position biomaterials as a promising platform for developing a functional “Vascular Band-Aid.” Such a band-aid, placed intraoperatively at the anastomotic sites, may provide immediate physical support while delivering sustained, localized and multimodal biological cues to preempt the pathological cascade of inflammation and intimal hyperplasia, offering a transformative strategy to proactively inhibit post-anastomotic complications.

To validate this concept, poly(L-lactide-co-ε-caprolactone) (PLCL) was selected as the substrate and applied to fabricate an aligned PLCL nanofibrous membrane using stable jet electrospinning [11, 12]. PLCL, a Food and Drug Administration (FDA)-approved biodegradable elastomer with a well-established safety profile, has recently gained recognition for vascular applications owing to its excellent biocompatibility and capacity to support cell growth [13–15]. The anisotropic architecture was specifically selected based on the principle of contact guidance, which provides structural cues that direct vascular cells to spread and migrate along the fiber orientation [16, 17], enhance cellular function through mechanotransduction [18], and promote ordered tissue regeneration with minimized disorganized scar, thereby facilitating restoration of vessel wall integrity at the suture line. To confer targeted therapeutic functions against anastomotic complications, a multifunctional surface coating was engineered as a biointerface to harness the synergistic properties of tannic acid (TA), copper ions (Cu^2+^) and poly-L-lysine (PLys). TA, a plant-derived polyphenol, exerts potent anti-inflammatory and cardioprotective effects through modulation of pathways including transforming growth factor-beta (TGF-β) and basic fibroblast growth factor [19]; Cu^2+^ promotes angiogenesis by enhancing endothelial cell (EC) migration and proliferation *via* sustained nitric oxide (NO)-catalytic activity [20, 21]; PLys, a cationic polypeptide, confers robust hemocompatibility by resisting platelet adhesion and activation, while its constituent lysine units possess intrinsic angiogenic properties [21, 22]. Notably, the TA–Cu^2+^ coordination complex coating formed on the patch surface can serve as a stable adhesive primer layer that enables sustained Cu^2+^ release and provides anchoring sites for subsequent grafting of amide-contained molecules. Thus, PLys was grafted onto this coating to generate a hierarchically structured surface that integrates complementary functionalities. This resulting multimodal platform is poised to mitigate inflammation, promote rapid vascular regeneration and inhibit complications at the anastomotic sites (Figure 1). Additionally, the cationic nature of PLys facilitates its interactions with negatively charged components of the extracellular matrix (ECM) [23], enhancing interfacial integration between the band-aid and the underlying vascular tissue. Interestingly, compared with conventional surface-engineering strategies (e.g. multistep covalent grafting, layer-by-layer assembly or plasma treatment), this method offers distinct practical advantages in terms of simplicity and universality: the entire modification avoids toxic organic solvents, complex catalysts or specialized equipment; and it can be readily adapted to other polymeric substrates and device geometries, not limited to PLCL-based patch, making it highly amenable to industrial scale-up and clinical translation. Collectively, the “Vascular Band-Aid” represents a comprehensive, localized therapeutic strategy with significant potential to proactively reduce postoperative complications and improve patency rates in vascular surgery.

**Figure 1:**
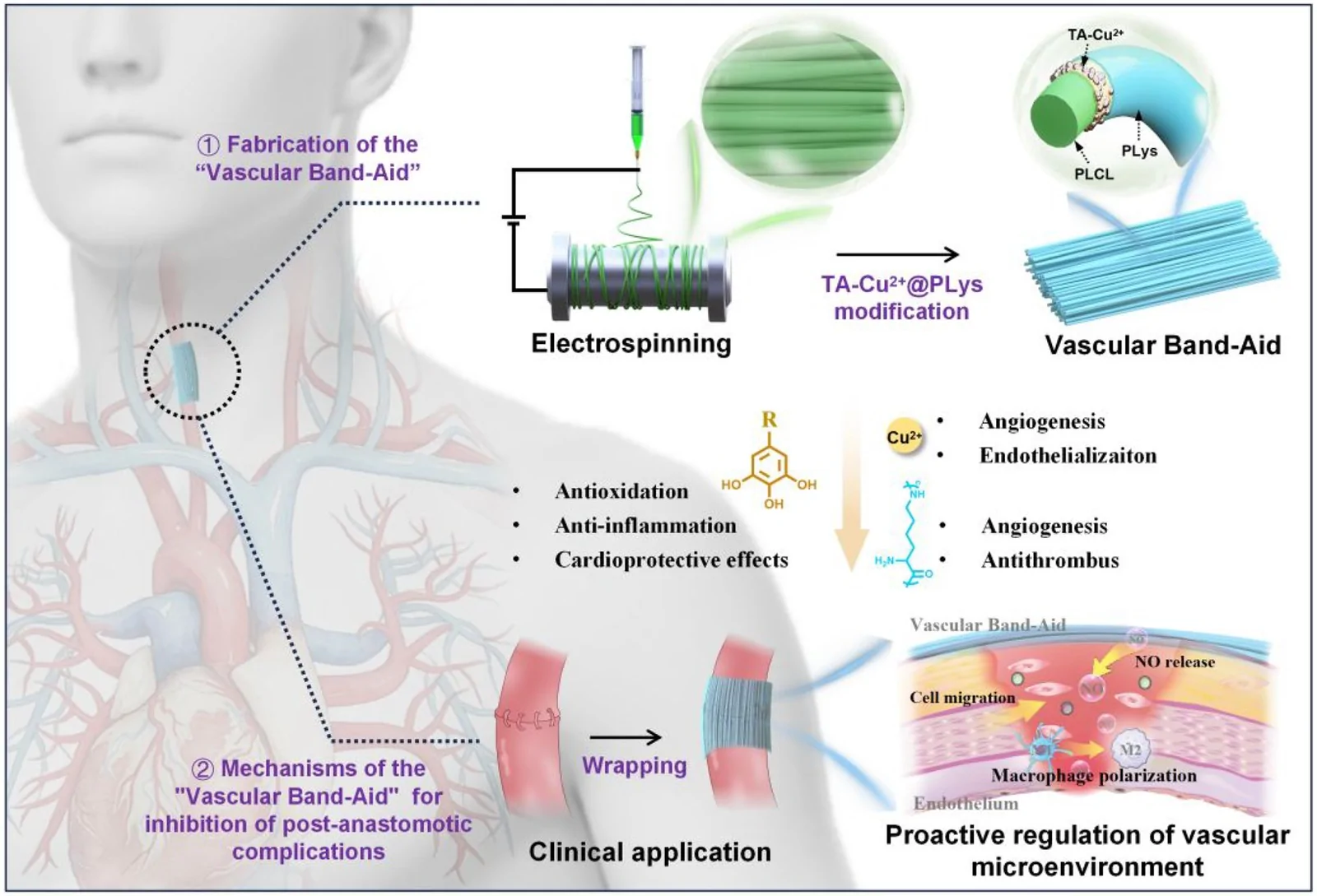
Schematic illustration of the PLCL-based “Vascular Band-Aid” for proactive inhibition of post-anastomotic vascular complications.

## Materials and methods

### Fabrication of the PLCL-based “Vascular Band-Aid”

#### Electrospinning of aligned PLCL nanofibers

Electrospinning (PS-3+, Qingdao Pansi Technology Co., Ltd) was employed to fabricate PLCL nanofibrous patch according to protocols described in our previous studies [15, 21, 22]. Briefly, a 5% (w/v) solution of PLCL (LA/CL = 50:50, intrinsic viscosity = 2.9 dL/g, Jinan Daigang Biomaterials, China) was prepared by solving PLCL in hexafluoroisopropanol (Macklin, China) for 12 h. Then, the electrospinning parameters were performed to fabricate the aligned nanofibers as follows: applied voltage of 5–6 kV, solution flow rate of 20 μL/min, collection distance of 20 cm and rotating drum of 1500 rpm. Aligned nanofibers were collected under ambient temperature and humidity (20–25°C, 25–30%). These as-spun membranes were subsequently vacuum-dried for 24 h.

#### Immobilization of TA–Cu^2+^ complexes onto PLCL nanofibers

A solution of tannic acid (TA; 8 mg/mL, Sigma, USA) and copper(II) chloride (0.1 mg/mL, Sinopharm Chemical Reagent, China) was prepared in 10 mM Tris-HCl buffer (pH 8.5, Shanghai Yuan Mu Biotechnology, China). The PLCL membranes were then immersed in this solution and shaken gently (100 rpm) at room temperature for 12 h under ambient atmosphere. Afterward, the samples were retrieved, thoroughly rinsed with deionized water and dried to obtain the TA–Cu^2+^-immobilized PLCL patch (denoted as TA–Cu^2+^).

#### Grafting of PLys molecules onto TA–Cu^2+^-immobilized PLCL patch

A 2 mg/mL solution of PLys (molecular weight: ∼5000, Macklin, China) was prepared in 10 mM Tris-HCl buffer. The TA–Cu^2+^-immobilized PLCL patch was then immersed in the PLys solution and shaken gently (100 rpm) at room temperature for 4 h. After the reaction, the samples were retrieved, thoroughly rinsed with deionized water and dried to obtain the final TA–Cu^2+^@PLys-modified PLCL patch (denoted as TA–Cu^2+^@PLys), i.e. the PLCL-based “Vascular Band-Aid.”

### Characterization of the PLCL-based “Vascular Band-Aid”

#### Surface morphology

To investigate the microscopic changes of PLCL nanofibers after TA–Cu^2+^ immobilization and PLys grafting, the samples were characterized by scanning electron microscope (SEM; Sigma 300, Zeiss AG, Germany) at an accelerating voltage of 0.02–30 kV. Prior to imaging, the samples were sputter-coated with gold to improve conductivity. Additionally, atomic force microscope (AFM; Dimension ICON, USA) was employed to directly visualize the morphological alterations and changes in fiber surface roughness.

#### Surface chemistry

The surface chemical composition of samples (i.e. PLCL, TA–Cu^2+^ and TA–Cu^2+^@PLys) was analyzed by Fourier transform infrared (FTIR; Thermo IS5, USA) spectroscopy over a wavenumber range of 4000–800 cm^−1^. Spectra were collected with an accumulation of 32 scans at a resolution of 4 cm^−1^. The surface elemental composition was further characterized by X-ray photoelectron spectroscopy (XPS; Thermo Scientific K-Alpha+, USA). The high-resolution C1s spectra were deconvoluted using a Gaussian–Lorentzian function with Origin 9.0 software.

#### Surface wettability

The surface wettability of PLCL, TA–Cu^2+^ and TA–Cu^2+^@PLys was characterized by water contact angle (WCA) measurements using an optical tensiometer (KRUSS DSA30S, Germany). Briefly, a 2 μL droplet of deionized water was deposited onto the nanofibrous membrane, and the changes in WCA were determined from real-time images of the droplet profile.

#### Water retention capacity

The water retention capacity of PLCL, TA–Cu^2+^ and TA–Cu^2+^@PLys was evaluated by a gravimetric method. Dried samples (1 cm × 1 cm) were first weighed to obtain the initial mass (*W*_0_). The samples were then fully immersed in ultrapure water and incubated with shaking at 37°C for 24 h to ensure thorough hydration. Afterward, the samples were removed, surface water was gently blotted and the final mass (*W*) was immediately measured. Finally, the water retention capacity was calculated using the following formula:


$$\mathrm{Water}\;\mathrm{retention}\;(\%)=\frac{W-W_{0}}{W_{0}}\times 100\%$$

#### Wet tensile property

The tensile property of the hydrated PLCL, TA–Cu^2+^ and TA–Cu^2+^@PLys samples was evaluated using a desktop tensile tester (Instron, USA). Prior to testing, all samples were equilibrated by immersion in deionized water for 24 h. Tensile tests were conducted at a constant tensile speed of 10 mm/min. The tensile strength of samples was calculated from the obtained stress–strain curves.

#### Surface zeta potential

The surface zeta potential of PLCL, TA–Cu^2+^ and TA–Cu^2+^@PLys was measured at 25°C using a SurPASS 3 Electrokinetic Analyzer (Anton Paar GmbH, Austria) *via* the streaming potential method. Measurements were performed at pH 4, 7 and 10 using a 1 mM potassium chloride solution as the background electrolyte.

#### Copper ion release

The cumulative release of Cu^2+^ from PLCL, TA–Cu^2+^ and TA–Cu^2+^@PLys was quantified over time using a commercial copper ion detection kit (Solarbio, China). Samples (1 cm × 1 cm) were incubated in 1 mL phosphate buffered saline (PBS) under constant agitation at room temperature. At predetermined intervals, the supernatant was collected and analyzed for Cu^2+^ amount according to the manufacturer’s protocol.

#### Protein adsorption property

The surface protein adsorption capacity of PLCL, TA–Cu^2+^ and TA–Cu^2+^@PLys was evaluated with a bicinchoninic acid (BCA) protein assay kit (Solarbio, China). Samples (15 mm diameter) were placed in a 24-well plate and incubated with 200 μL of bovine serum albumin (BSA; 50 μg/mL, Sangon Biotech, China) solution at room temperature for 2 h. After incubation, the samples were rinsed thoroughly three times with deionized water. Subsequently, 300 μL of the BCA working reagent was added and the samples were incubated at 37°C for 30 min. For quantification, 100 μL of the resulting solution was transferred to a 96-well plate, and the absorbance at 562 nm was measured using a microplate reader (Thermo Fisher Scientific, USA) to determine the adsorbed BSA content.

### *In vitro* biofunctional assessment of the PLCL-based “Vascular Band-Aid”

#### Cell culture and seeding

Human umbilical vein endothelial cells (HUVECs; ScienCell, USA) were cultured in a commercial endothelial cell growth medium (PCM-H-040-PRO, ZQXZbio, China), supplemented with 5% fetal bovine serum, 1% penicillin/streptomycin and 1% endothelial cell growth factor supplement. The culture medium was changed every 48 h. Prior to cell seeding, the samples were sterilized by exposure to ultraviolet light for 6 h, followed by immersion in 75% ethanol for 2 h. The samples were then rinsed three times with PBS. Subsequently, the sterilized samples were placed in 24-well culture plates and preconditioned by incubation in culture medium at 37°C overnight. HUVECs at ∼90% confluence were harvested and seeded onto the pretreated samples for subsequent experiments.

#### Biocompatibility

To assess the hemocompatibility of the samples, fresh rat blood was collected and centrifuged at 5000 rpm for 5 min to obtain a red blood cell suspension. The samples (1 cm × 1 cm) were then cocultured with the red blood cell suspension at 37°C for 1 h. Subsequently, the supernatant was taken to a 96-well culture plate, and the absorbance was measured at 545 nm using a spectrophotometer. The hemolysis rate was then calculated. A solution containing 2% red blood cell suspension and 98% physiological salt was used as the negative control, while a solution containing 2% red blood cell suspension and 98% ddH_2_O was used as the positive control.

To evaluate cytocompatibility, HUVECs were seeded onto the samples in 24-well plates at a density of 4 × 10^4^ cells per well. After 24 h of culture, cell viability was observed and quantified using a commercial LIVE/DEAD viability/cytotoxicity assay kit (Beyotime, China). Cell viability was calculated based on the ratio of live (green) to dead (red) cells.

#### Cell affinity

To assess cell affinity, HUVECs were seeded onto the samples in 24-well plates at a density of 4 × 10^4^ cells per well. For initial adhesion analysis, the cells were cultured for 30 min, fixed with 4% paraformaldehyde (Biosharp, China) in PBS for 30 min and permeabilized with 0.2% Triton X-100 (Solarbio, China) for 5 min. After rinsing thoroughly with PBS, the actin cytoskeleton was stained with TRITC-phalloidin (1:200 dilution, Yeasen Biotechnology, China) in the dark at room temperature for 30 min, followed by nuclear staining with DAPI (1:1000 dilution, Yeasen Biotechnology, China) for 10 min. The adherent cells were imaged using an inverted fluorescence microscope, and the number of cells was quantified using ImageJ software. For cell spreading analysis, cells were incubated for 24 h postseeding, stained using the same protocol and imaged. The average cell spreading area was quantified using ImageJ software.

To evaluate cell proliferation, a Cell Counting Kit-8 (CCK-8, MeilunBio, China) assay was performed after 1, 3 and 5 days of culture. Briefly, 500 μL of a 10% CCK-8 working solution in fresh medium was added to each well. The plates were incubated at 37°C for 1.5 h. Subsequently, 100 μL of the solution from each well was transferred to a 96-well plate, and the absorbance at 450 nm was measured using a microplate reader (BioTek, USA). Cell proliferation was analyzed based on the recorded absorbance values.

#### Cytoprotective effects against inflammatory activation

To evaluate the cytoprotective effects of samples, oxidative stress was induced in HUVECs using hydrogen peroxide (H_2_O_2_) as an inflammatory microenvironment simulator, and intracellular reactive oxygen species (ROS) levels were quantified. Briefly, HUVECs were seeded onto the samples in 24-well plates at a density of 4 × 10^4^ cells per well and cultured for 24 h. The culture medium was then replaced with fresh complete medium containing 400 μM H_2_O_2_ (Macklin, China), and the cells were incubated for an additional 24 h. Afterward, intracellular ROS was stained using a commercial assay kit (MeilunBio, China) according to the manufacturer’s protocol. Fluorescence imaging was performed using an inverted microscope, and the mean fluorescence intensity, corresponding to ROS levels, was quantified from the obtained images using ImageJ software.

#### Endothelial cell functions

To investigate the gene expression profile of HUVECs, cells were seeded onto the samples in 6-well plates at a density of 5 × 10^5^ cells per well and cultured for 24 h. Total RNA was extracted using a commercial RNA isolation kit (Biozol, China) and reverse transcribed into cDNA using the FastKing RT Kit (with gDNase; Tiangen, China). Reverse transcription-quantitative polymerase chain reaction (RT-qPCR) was performed using a SuperReal PreMix Plus (SYBR Green) kit (Tiangen, China) on an ABI Prism 7500 Sequence Detection System (ABI, Singapore). The mRNA expression of genes associated with cell–matrix interaction, such as *Integrin β1* and *myosin light chain kinase* (*MLCK)*, and cell differentiation, such as *platelet endothelial cell adhesion molecule-1* (*CD31*) and *vascular endothelial cadherin* (*VE-cadherin*) was quantified. All the target gene expression was normalized to the endogenous reference gene, *glyceraldehyde 3-phosphate dehydrogenase* (*GAPDH*). Primer sequences are provided in Table S1.

### *In vivo* animal experiments

#### Establishment of a rabbit carotid intima hyperplasia model

All animal procedures were approved by the Animal Ethics Committee of the University of Health and Rehabilitation Sciences (Approval No. KFDX: No. 2026-4007) and conducted in accordance with the guidelines of the Chinese Institutional Animal Care and Use Committee. To evaluate the ability of the “Vascular Band-Aid” to prevent postoperative complications and promote vascular repair, an intimal hyperplasia model was established in the rabbit carotid artery, followed by wrapping the “Vascular Band-Aid” around the suture site. Sixteen healthy male New Zealand White rabbits (6 weeks old, 1.8–2.0 kg, Qingdao Kangda Aibo Biotechnology, China) were randomly divided into two groups: a control group (pristine intima hyperplasia model) and an experimental group (treated with the TA–Cu^2+^@PLys). Anesthesia was induced *via* intramuscular injection of 0.8–1.2 mL Zoletil50 (Virbac, France). Then, the neck region was shaved and disinfected, and a midline incision was made to expose the right common carotid artery. The artery was clamped, transected and the endothelium was mechanically injured. The two ends were anastomosed using 9-0 nylon sutures under a surgical microscope. The anastomotic site was then wrapped with TA–Cu^2+^@PLys (or left untreated in the control group). Vascular clamps were removed to restore blood flow, and the skin incision was closed with 4-0 nylon sutures. Animals were housed individually and allowed free access to food and water throughout the study.

#### Histological staining

To evaluate postoperative complications, tissue specimens were harvested at 1 W and 4 W, fixed in 4% paraformaldehyde, paraffin-embedded and sectioned at 5 μm thickness. Sections were stained with hematoxylin and eosin (H&E; Servicebio, China) and Masson’s trichrome (Servicebio, China). Bright-field imaging was performed using a microscope-coupled camera system (NIKON DS-U3, Japan). The collagen deposition was quantified from the obtained Masson images.

Immunohistochemical (IHC) staining was performed to assess the expression of pro-inflammatory marker tumor necrosis factor-α (TNF-α) and anti-inflammatory marker TGF-β in the rabbit specimens. Briefly, tissue sections were treated with 1% H_2_O_2_ in methanol for 20 min to quench endogenous peroxidase activity. After antigen retrieval and blocking, sections were incubated overnight at 4°C with primary antibodies against TNF-α antibody (ABclonal, A21265, China) or TGF-β1 (ABclonal, A25313, China). Following washing, sections were incubated with an HRP-conjugated secondary antibody (Servicebio, China) at room temperature for 2 h. Signal development was performed using a 3,3′-diaminobenzidine substrate for 10 min, followed by counterstaining with hematoxylin. Stained sections were imaged, and the immunostaining intensity was quantitatively analyzed using ImageJ software.

Immunofluorescence staining was performed to assess vascular repair and regenerative capacity. Tissue sections were blocked with 10% donkey serum (Servicebio, China) at room temperature for 30 min. Subsequently, sections were incubated overnight at 4°C with the following primary antibodies diluted in blocking buffer: CD31 (ABclonal, A19014, China) for ECs, alpha-smooth muscle actin (α-SMA; ABclonal, A4064, China) for smooth muscle cells (SMCs), proliferating cell nuclear antigen (PCNA; ABclonal, A0264, China) and Integrin β1 (ABclonal, A21234, China) for cell proliferation and migration. After washing, sections were incubated for 2 h at room temperature with the corresponding secondary antibodies: CY3-labeled IgG (Servicebio, GB21303, China) or FITC-labeled IgG (Servicebio, GB22403, China). Nuclei were counterstained with DAPI for 10 min. Fluorescence imaging was performed using a Nikon DS-U3 microscope system. The fluorescence intensity of specific markers was quantified using ImageJ software.

### Statistical analysis

Data are presented as the mean ± standard deviation (SD) of three or more independent experiments. Origin software was used for one-way analysis of variance (ANOVA) followed by Tukey’s test for comparisons. A *P*-value < 0.05 was considered statistically significant (NS: no significant difference, *P < 0.05, **P < 0.01, ***P < 0.001).

## Results and discussion

### Engineering a proactive “Vascular Band-Aid” through stepwise modification of TA–Cu^2+^ complexes and PLys molecules

Vascular patches require compliance matching to accommodate pulsatile distension. PLCL combines the strength of poly(L-lactic acid) (PLLA) with the ductility of poly(ε-caprolactone) (PCL), imparting rubber-like elasticity that maintains structural integrity and flexibility [24]. This unique combination positions PLCL as a superior alternative to PLLA, PCL and nondegradable polymers for vascular patch applications. TA–Cu^2+^ modification provides anti-inflammatory and proangiogenic activities, but it lacks sufficient hemocompatibility and antithrombogenicity. Conversely, PLys grafting enhances antithrombogenicity and cell adhesion but fails to address the persistent oxidative/inflammatory microenvironment. In this context, a stepwise modification with TA–Cu^2+^ complexes was performed onto PLCL patch, wherein the PLys-rich outer layer ensures immediate blood compatibility and rapid cell adhesion, while the underlying TA–Cu^2+^ reservoir sustains local anti-inflammatory and provascularization signals over time. This complementary interplay enables multitarget intervention against the pathological cascade, such as inflammation, thrombosis and dysregulated repair. For the mechanisms involved in the stepwise modification, the abundant phenolic hydroxyl groups of TA can undergo autoxidation to yield *o*-quinones under ambient or mild oxidative conditions [25], and then form a dense hydrogen-bonding network with the ester oxygen atoms and terminal hydroxyl groups of the PLCL matrix. Concurrently, the hydrophobic interactions, further stabilized by π–π stacking, bring the aliphatic carbon chains of PLCL into close proximity with the aromatic ring regions of TA, thereby establishing a stable noncovalent binding system. In parallel, the pyrogallol moieties of TA undergo self-polymerization with unoxidized pyrogallol groups on adjacent TA molecules, generating a covalently crosslinked polyphenolic network that ensures robust inter-TA connectivity [19]. Moreover, TA acts as a bridging agent to facilitate chelation of Cu^2+^ onto the PLCL nanofibers through the classical polyphenol–metal ion interaction [26], while providing abundant anchor sites (oxidized *o*-quinone structures) for binding the –NH_2_ groups of PLys through Michael addition and Schiff-base reactions (Figure 2A).

**Figure 2:**
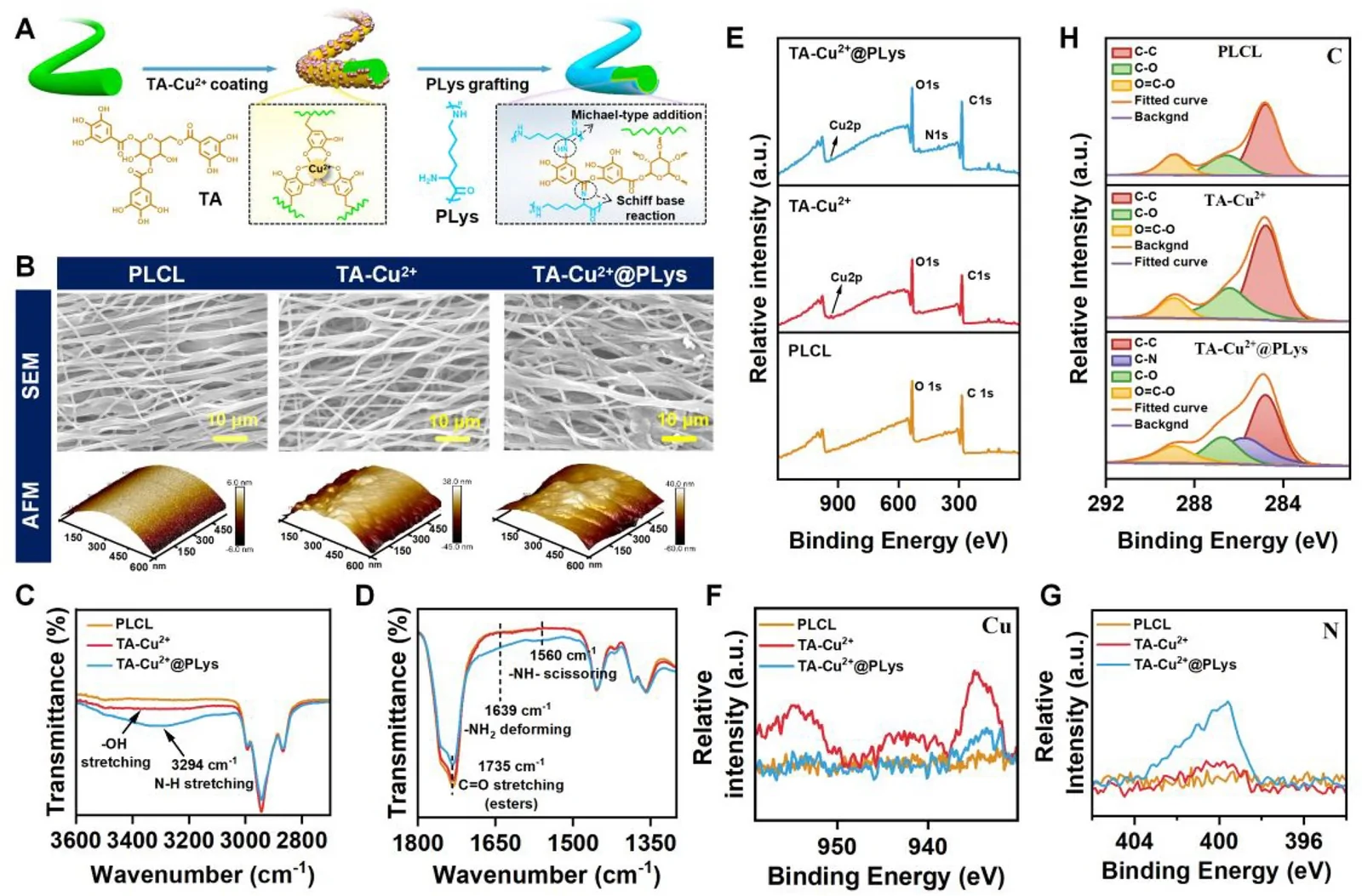
Morphological and chemical characterizations of the “Vascular Band-Aid”: (**A**) schematic illustration of stepwise modification with TA–Cu^2+^ complexes and PLys molecules; (**B**) SEM and AFM images; (**C** and **D**) the partial region enlarged FTIR spectra; (**E**) XPS survey spectra; (**F** and **G**) XPS spectra of Cu2p and N1s; (**H**) XPS spectra for the deconvolution of C1s peaks.

SEM revealed that the stepwise modification with TA–Cu^2+^ complexes and PLys molecules altered the overall fiber morphology of the “Vascular Band-Aid” (Figure 2B). The average fiber diameter increased from 1.01 ± 0.16 μm (PLCL) to 1.07 ± 0.12 μm (TA–Cu^2+^) and further to 1.15 ± 0.22 μm (TA–Cu^2+^@PLys) (Figure S1). This indicates that the coating thickness of TA–Cu^2+^ is ∼30 nm, consistent with previous reports [21, 27] and increased to about 70 nm after PLys grafting. AFM showed that pristine PLCL fibers exhibited a smooth, clean surface with a roughness (Ra) of 3.64 ± 1.72 nm. By contrast, the TA–Cu^2+^ coating resulted in a rough surface with nanoaggregates (Ra: 11.39 ± 3.69 nm; Figure S2), attributable to the accumulation of self-polymerized TA nanoparticles. Following PLys grafting, the surface roughness increased to 13.37 ± 1.03 nm, which correlated well with the size of PLys molecules and resulted from the TA-mediated anchoring of PLys. FTIR spectroscopy was employed to analyze changes in surface functional groups of the “Vascular Band-Aid” (Figure S3A). Compared with PLCL, the appearance of the broad peak at ∼3500–3200 cm^−1^ was assigned to the O–H stretching of phenolic hydroxyl groups in TA (Figure 2C), confirming successful TA coating. Upon PLys introduction, the absorption intensity at 3294 cm^−1^ increased, indicative of N–H stretching from primary amine vibrations [21]. Additionally, asymmetric absorption peaks corresponding to –NH– (scissoring vibration) and –NH_2_ (deforming vibration) further confirmed successful PLys grafting onto the PLCL nanofibers (Figure 2D). XPS was performed to further investigate the functionalization process (Figure 2E). Compared with PLCL, the appearance of a Cu2p peak in TA–Cu^2+^ revealed successful Cu^2+^ deposition (Figure 2F). After PLys modification, the intensity of the Cu2p peak decreased, which can be attributed to signal attenuation caused by the PLys molecule coverage. In addition, the appearance of an N1s peak provides complementary evidence for successful PLys grafting (Figure 2G). The C1s core-level spectrum was curve-fitted into four peaks at 288.5 eV (O = C–O), 286.2 eV (C–O), 285.1 eV (C–N) and 284.5 eV (C–C) (Figure 2H). Compared with PLCL, the proportion of C–O bonds in TA–Cu^2+^ increased markedly (Table S2), consistent with TA deposition. After PLys grafting, a C–N peak emerged as expected. Notably, the amount of –NH– groups (generated by Michael-type addition) exceeded that of –N = groups (generated by Schiff-base reaction), indicating that PLys grafting onto the TA–Cu^2+^ coating occurred preferentially through Michael addition (Figure S3B). Importantly, the content of –NH_2_ groups remained higher than that of both –NH– and –N = groups, suggesting that a substantial number of primary amines were preserved after PLys grafting, which is a feature favorable for cell recognition and biological response [28].

### Surface engineering with –NH_2_ and phenolic hydroxyl groups imparts hydrophilicity, positive charge and protein adsorption to the “Vascular Band-Aid”

The surface physicochemical properties (e.g. wettability, stiffness and surface charge) of implants initially influence protein adsorption through early surface processes, and subsequently control cell–material interactions to mediate therapeutic effects after implantation [29]. Hydrophobic surfaces can alter the conformation of adsorbed proteins, impairing cell recognition of specific binding sites [30]. This leads to slow or ineffective cell adhesion and spreading, ultimately causing cell death or growth arrest. By contrast, moderately hydrophilic surfaces (typically with WCA between 40° and 70°) preserve the native conformation of adhesive proteins, thereby promoting cell adhesion and spreading *via* focal adhesion remodeling [31, 32]. As shown in Figure 3A and B, the WCA of PLCL exceeded 90°, indicating a hydrophobic surface. After stepwise modification with TA–Cu^2+^ and PLys, the WCA of the “Vascular Band-Aid” decreased sharply to ∼40°, primarily attributable to increased surface roughness and the retention of abundant hydrophilic groups (such as –NH_2_ and phenolic hydroxyl groups). This trend was confirmed by water retention measurements (Figure 3C), in which the water retention capacities of TA–Cu^2+^ and TA–Cu^2+^@PLys were significantly higher than that of PLCL.

**Figure 3:**
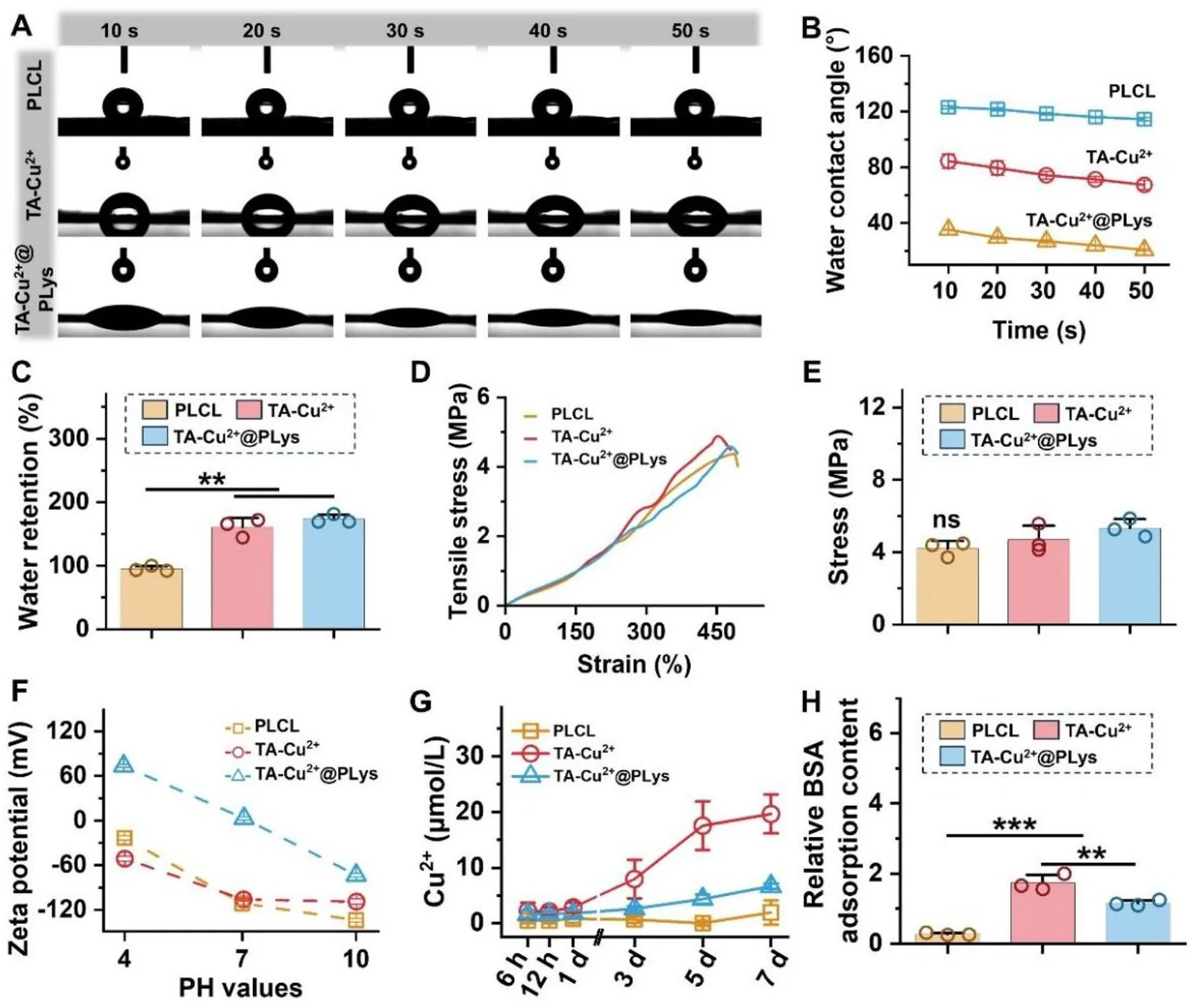
Physicochemical properties of the proactive “Vascular Band-Aid”: (**A** and **B**) surface wettability; (**C**) water retention; (**D** and **E**) wet tensile properties and tensile strength; (**F**) zeta potential; (**G**) Cu^2+^ release within 7 days; (**H**) BSA adsorption capacity. **P < 0.01, ***P < 0.001.

Substrate stiffness physically influences cell fate and tissue development through mechanotransduction mechanisms [33]. Possibly owing to the negligible coating thickness relative to fiber diameter, the stepwise deposition of TA–Cu^2+^ and PLys had no significant effect on the wet tensile properties of the substrate (Figure 3D and E). Surface charge influences early cell–material interactions by modulating protein adsorption and subsequent integrin-mediated signaling, which in turn affects cell adhesion, spreading and proliferation [34, 35]. In general, positively charged surfaces promote cell proliferation and spreading and activate signaling cascades involved in the immune system and regenerative responses to biomaterials [34]. Zeta potential measurements revealed a pH-dependent change in surface charge for all samples (Figure 3F), attributable to the protonation/deprotonation behavior of their terminal functional groups [22]. Specifically, PLCL surfaces exhibit a negative zeta potential under physiological conditions, which can be attributed to the ester linkages in the polymer backbone and the limited number of terminal carboxyl groups at chain ends [36]. Upon stepwise modification with TA–Cu^2+^ complexes (involving negatively charged phenolic hydroxyl groups) and PLys molecules (containing the abundant positively charged –NH_2_ groups), higher zeta potential surface yields and tends to be favorable for cell growth.

Cu^2+^, which exhibit glutathione peroxidase-like activity that catalyzes *in situ* NO release from endogenous S-nitrosothiols [21], were chelated within the TA-based polyphenolic network. TA–Cu^2+^ showed slow Cu^2+^ release over a period of 7 days (Figure 3G). Notably, PLys grafting reduced the Cu^2+^ release rate because of the enhanced network stability conferred by PLys incorporation. This release profile suggests that the “Vascular Band-Aid” has the potential to provide prolonged bioactivity at the anastomotic site, which may contribute to the modulation of cell behavior and tissue remodeling over the course of healing. To directly evaluate the protein adsorption capacity of the “Vascular Band-Aid”, BSA adsorption was quantitatively analyzed (Figure 3H). Compared with PLCL, TA–Cu^2+^ improved protein adsorption capacity owing to the introduction of abundant phenolic hydroxyl groups. However, subsequent modification with PLys molecules consumes some of the phenolic hydroxyl groups, thus leading to a decrease in protein adsorption capacity. These findings indicate that surface engineering with TA–Cu^2+^ and PLys enhances the protein adsorption capacity of the Vascular Band-Aid, and preserves the active conformation of adsorbed proteins through high hydrophilicity. This property, together with its positively charged interface [37], synergistically promotes cell adhesion.

### The “Vascular Band-Aid” featuring a bioactive interface enables proactive biocompatibility and enhanced cell affinity

Biocompatibility, originally defined as the ability of biomaterials to elicit an appropriate host response in a specific application [38], is an essential requirement for the clinical translation of the “Vascular Band-Aid”. In the context of vascular implants, biocompatibility is broadly categorized into hemocompatibility and cytocompatibility. Hemocompatibility refers to the material’s tolerance toward blood and primarily relates to thrombogenic and thromboembolic complications induced by biomaterial interfaces [39], representing a prerequisite for their short- and long-term applications. Hemolysis assays revealed that all samples exhibited good hemocompatibility (Figure 4A), with hemolysis rates well below the acceptable 5% threshold for blood-contacting materials [22] and no significant hemolytic reactions. Notably, TA–Cu^2+^@PLys supported a slight but negligible reduction in hemolysis ratio and induced less clot formation (Figure S4), possibly owing to the mild shielding effect conferred by the amine-rich PLys. This shielding preserves the conformational integrity of adsorbed proteins (e.g. fibrinogen) and thereby inhibits platelet adhesion and activation *via* selective blockade of such GPIIb/IIIa platelet receptors [22].

**Figure 4:**
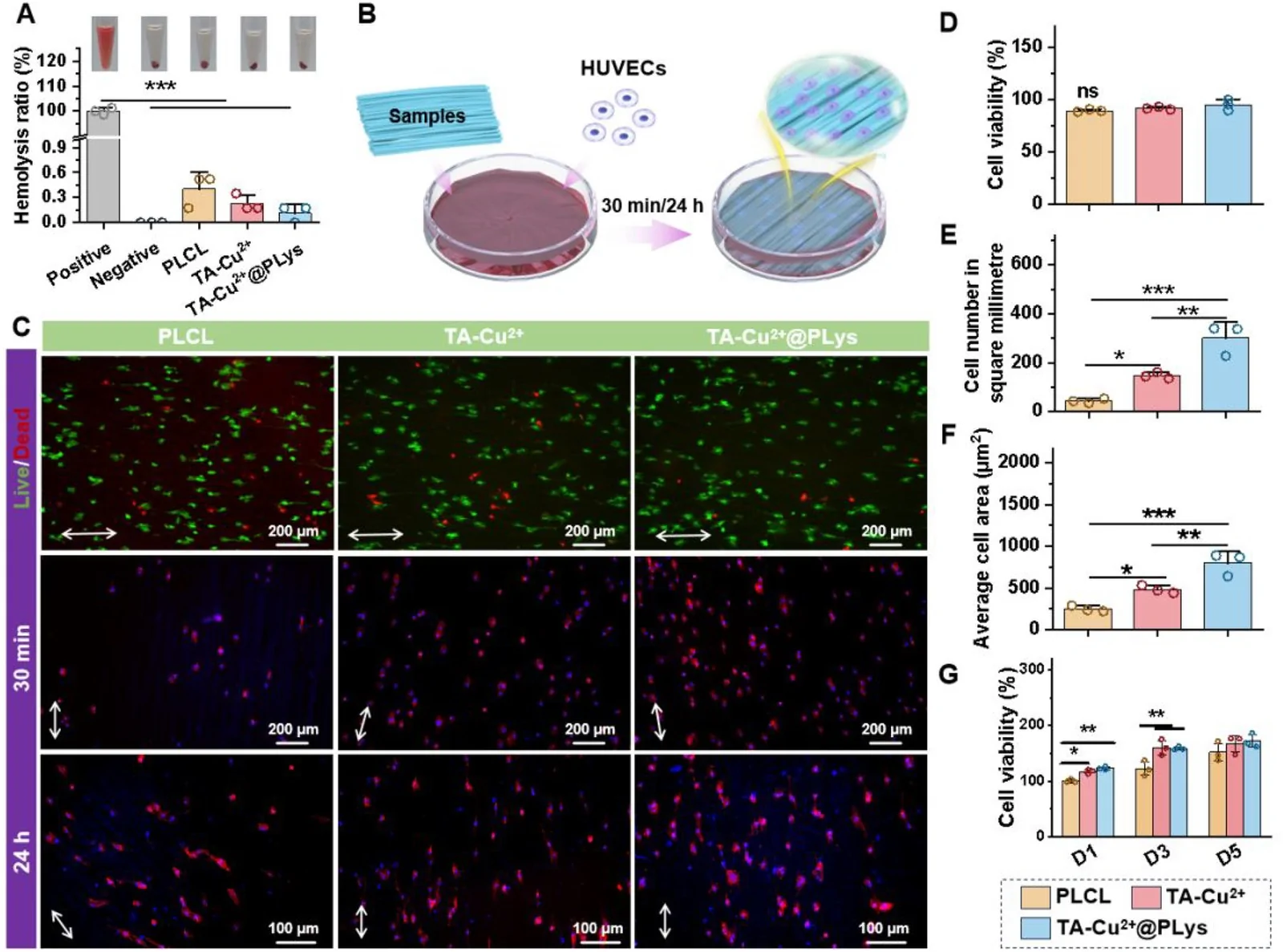
Biocompatibility and cell affinity of the “Vascular Band-Aid”: (**A**) hemolysis ratio; (**B**) schematic illustration of cytocompatibility assay; (**C**) cell viability for 24 h, adhesion for 30 min and spreading for 24 h. The white double-headed arrows indicate the direction of fiber orientation; (**D**–**F**) quantification of cell viability, adhered cell number and spreading cell area from image **C**; (**G**) cell proliferation. *P < 0.05, **P < 0.01, ***P < 0.001.

Cytocompatibility describes the ability of biomaterials to interface with living cells, governing the cellular or tissue response that underlies therapeutic efficacy [40]. To assess the cytocompatibility of the “Vascular Band-Aid,” HUVECs were seeded on samples for 30 min or 24 h (Figure 4B). Within 24 h, all samples supported >95% cell viability without significant cell death (Figure 4C and D), confirming favorable cell affinity. Notably, stepwise modification with TA–Cu^2+^ complexes and PLys molecules exerted a stimulatory effect on cell adhesion and spreading (Figure 4E and F). This effect is attributed to a synergistic combination of factors: the polyphenolic groups of TA facilitating binding to cell membrane proteins [41], cationic amino acid residues forming charge interactions with anions on the cell surface [42], and increased surface roughness providing a more favorable physical topography for cell spreading [43]. These findings indicate that TA–Cu^2+^@PLys enhances cell affinity and supports ECs growth. Changes in cell morphology generally influence the switch between survival and apoptosis by activating key signaling pathways, such as Rho family GTPases and mitogen-activated protein kinase [44]. To further evaluate HUVEC proliferation, cells were seeded on samples for 1, 3 and 5 days. Compared with PLCL, TA–Cu^2+^@PLys significantly promoted cell proliferation while maintaining high viability (Figure 4G). These findings demonstrated that the engineered “Vascular Band-Aid” effectively enhanced HUVEC growth, enabling proactive biocompatibility and improved cell affinity.

### The “Vascular Band-Aid” exhibits both antioxidant activity and enhanced endothelial cell differentiation through MLCK-mediated mechanotransduction

In chronic disease settings, uncontrolled or persistent inflammation is a critical driver of complications at vascular anastomotic sites. Excessive inflammation activates the coagulation cascade and induces a contractile-to-proliferative phenotypic switch in SMCs, leading to thrombosis or abnormal intimal hyperplasia at the anastomotic sites [45, 46]. Furthermore, it impairs EC migration and disrupts ECM homeostasis, constraining vascular repair and remodeling [47]. Given that ROS signaling contributes to maladaptive inflammatory responses [48], we employed H_2_O_2_ treatment to mimic the inflammatory microenvironment and assessed ROS expression in HUVECs to evaluate the antioxidant capacity of the “Vascular Band-Aid”. As shown in Figure 5A, cells cultured on PLCL exhibited a marked oxidative response, with elevated ROS levels upon H_2_O_2_ exposure. Notably, modification with the TA–Cu^2+^@PLys coating significantly improved the free radical scavenging capacity of the “Vascular Band-Aid” (Figure S5), thus substantially reducing cellular ROS expression (Figure 5B), indicating alleviation of inflammation-induced cytotoxicity and demonstrating its antioxidant, anti-inflammatory properties.

**Figure 5:**
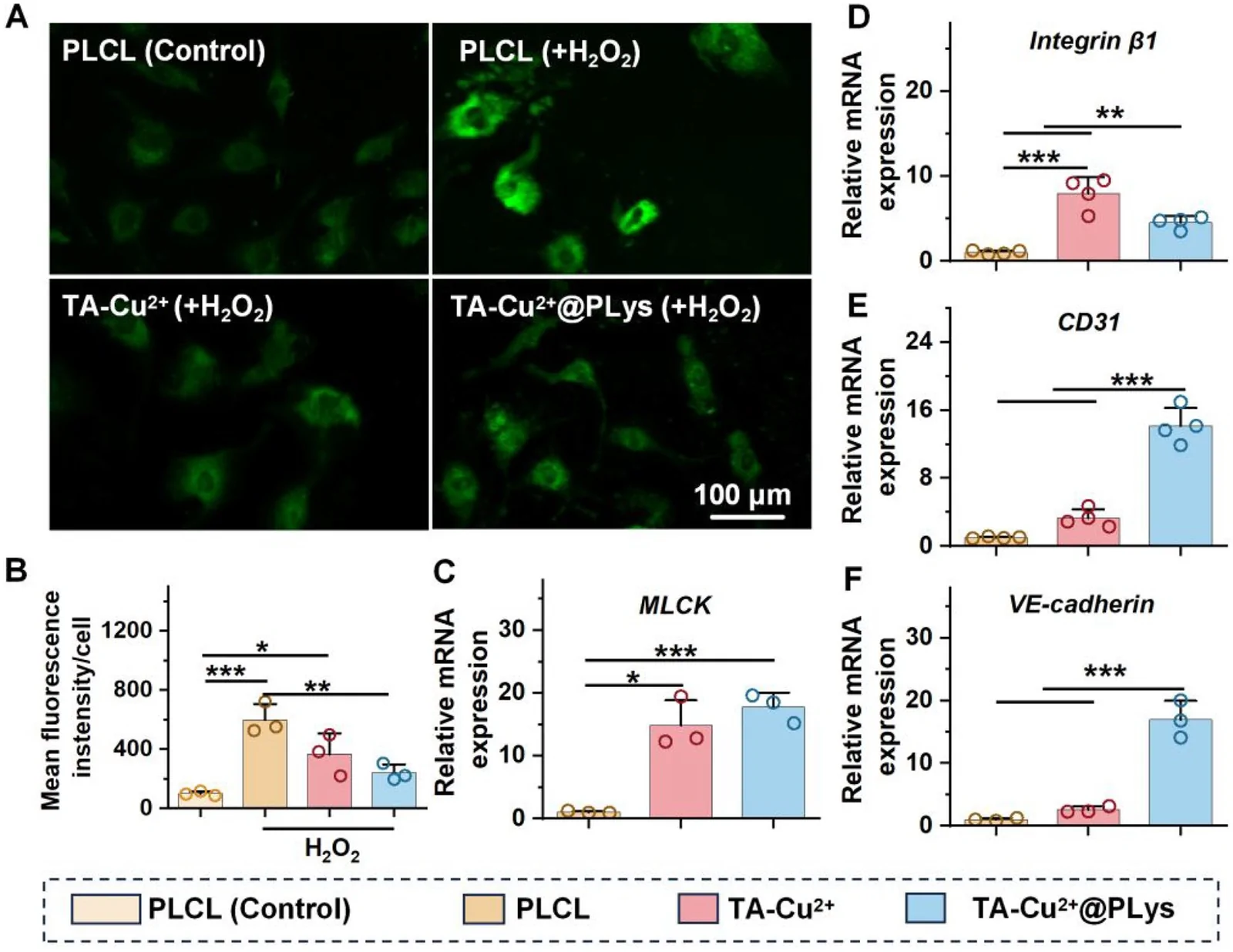
Antioxidant activity and prodifferentiation effects of the “Vascular Band-Aid” on HUVECs: (**A** and **B**) intracellular ROS expression in HUVECs exposed to H_2_O_2_; (**C**–**F**) RT-qPCR analysis of *MLCK*, *Integrin β1*, *CD31* and *VE-cadherin* expression levels normalized to GAPDH. *P < 0.05, **P < 0.01, ***P < 0.001.

The expression of genes associated with endothelial function was further examined, including *MLCK*, *Integrin β1*, *CD31* and *VE-cadherin*. Integrins serve as the principal molecular link between cells and ECM/biomaterials. By recruiting intracellular adaptors and ECM signaling proteins, integrins assemble into large adhesion complexes that mediate bidirectional signaling across cell–matrix interfaces through MLCK-dependent cytoskeletal contraction and rearrangement (mechanotransduction) [49]. This process regulates diverse cellular functions, including gene expression, proliferation, survival, polarity, differentiation and crosstalk with other receptor systems. Compared with PLCL, TA-Cu^2+^ modification upregulated both *MLCK* and *Integrin β1* expression (Figure 5C and D), indicating enhanced integrin-mediated cell–matrix interactions. Following PLys grafting, *Integrin β1* expression was reduced, whereas *MLCK* expression remained unchanged relative to TA-Cu^2+^. This finding suggests that PLys introduction diverts integrin β1-mediated cell–matrix signaling pathways while leaving MLCK-mediated cytoskeletal contraction and rearrangement intact. CD31 and VE-cadherin, which are specific surface markers of ECs, directly reflect HUVEC maturity and proangiogenic capacity. Compared to PLCL and TA–Cu^2+^, the increased expression of *CD31* and *VE-cadherin* in TA–Cu^2+^@PLys indicated that PLys grafting efficiently induced HUVEC differentiation toward a mature phenotype (Figure 5E and F). This effect arises possibly from electrostatic interactions between the positively charged amino residues of PLys and the negatively charged cell membrane surface, which in turn activate protein kinase C-related signaling pathways to regulate cell responses [50]. Collectively, these findings demonstrate that the “Vascular Band-Aid” exhibits both antioxidant activity and enhanced EC differentiation through cell–matrix interactions and MLCK-mediated mechanotransduction.

### The “Vascular Band-Aid” effectively inhibits complications and promotes vascular repair at anastomotic sites

Owing to its combined anti-inflammatory, proendothelializing and antithrombotic activities, TA–Cu^2+^@PLys, referred to as the “Vascular Band-Aid”, is expected to suppress post-anastomotic complications and expedite vascular repair. To assess its *in vivo* efficacy, an artery intimal hyperplasia model was established at the anastomotic sites (Figure 6A). At 1 week postsurgery (Figure 6B), the control group exhibited pronounced vascular occlusion resulting from endothelial injury-induced thrombosis. In contrast, vessels treated with TA–Cu^2+^@PLys remained patent. Masson’s trichrome staining demonstrated extensive erythrocyte filling within control vessels, confirming thrombus formation. Closer examination disclosed small amounts of collagen within the thrombus, a characteristic feature of thrombosis accompanied by early intimal hyperplasia. In the TA–Cu^2+^@PLys group, the vascular lumen appeared clean and continuous cellular coverage was observed along the inner surface of the suture site, indicative of positive endothelial remodeling. At 4 weeks postsurgery (Figure 6C), the TA–Cu^2+^@PLys group continued to exhibit patent vessels, with more mature and continuous collagen formed at the suture site and integrated seamlessly with the adjacent tissue. In the control group, although the thrombus area had decreased, collagen content within the residual thrombus had substantially increased and matured, indicating exacerbated intimal hyperplasia [51].

**Figure 6:**
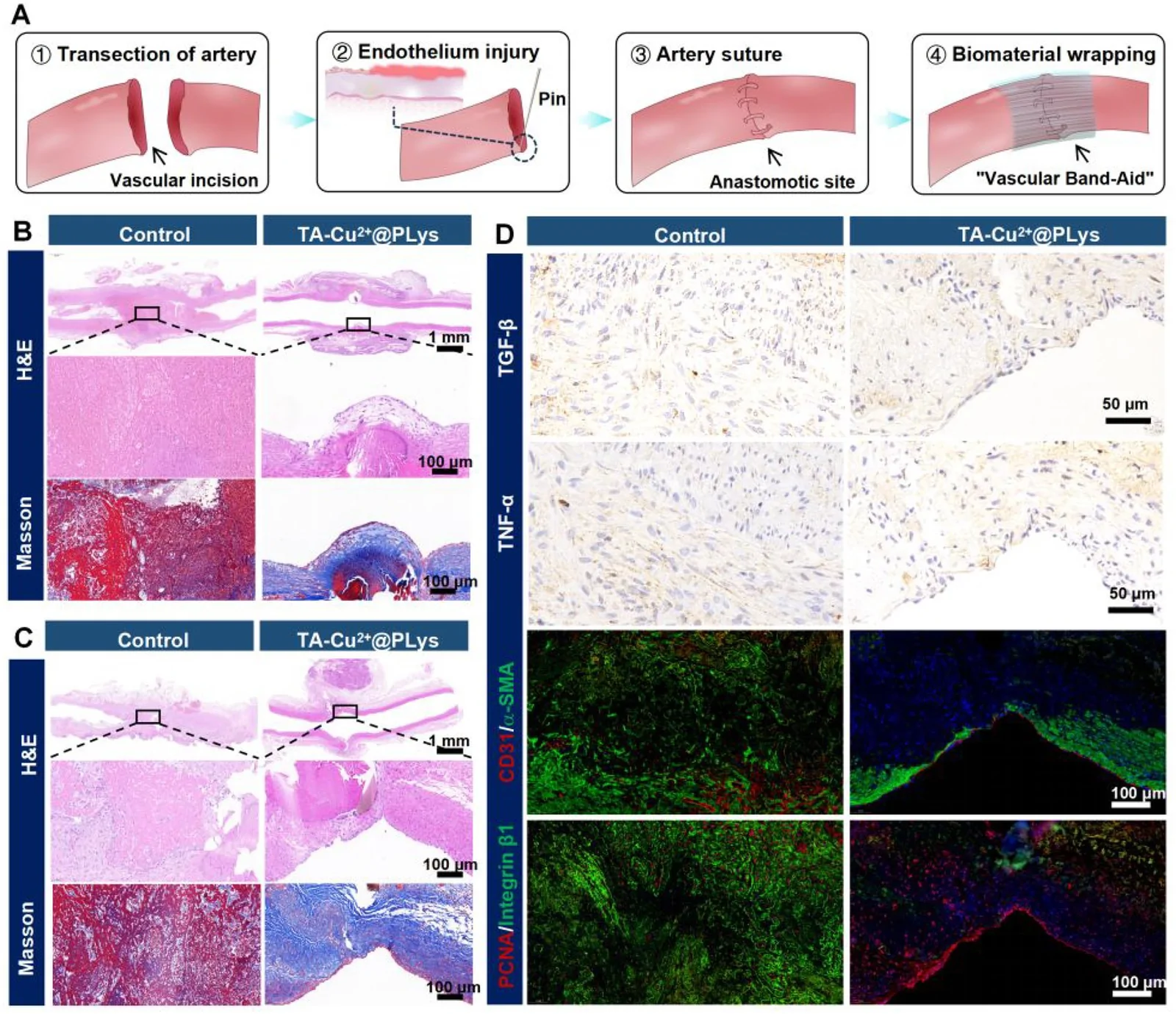
Effects of “Vascular Band-Aid” on inhibiting post-anastomotic complications: (**A**) schematic illustration of the carotid intima hyperplasia model; (**B**) H&E and Masson’s trichrome staining of samples at 1 W; (**C**) H&E and Masson’s trichrome staining of samples at 4 W; (**D**) immunofluorescence and immunohistochemical staining of samples at 4 W.

IHC staining for the proinflammatory marker TNF-α and the anti-inflammatory marker TGF-β was performed to evaluate inflammatory responses (Figure 6D). All groups exhibited marked expression of both TNF-α and TGF-β, indicating pronounced inflammatory activation [52, 53]. In comparison, the TGF-β/TNF-α ratio was elevated in the TA–Cu^2+^@PLys group relative to control (Figure S6), suggesting a shift toward an anti-inflammatory state. Effective inhibition of anastomotic complications requires EC regeneration and functional maturation of SMCs. In the TA–Cu^2+^@PLys group, continuous CD31-positive cell coverage was observed along the luminal surface (Figure 6D), indicating positive endothelial reconstruction [54]. Staining for α-SMA, a marker of SMCs [55], revealed active remodeling of a regularly arranged and ordered smooth muscle layer, which contributes to mechanical stability at the anastomotic sites. In contrast, the control group showed abundant yet randomly distributed α-SMA-positive cells within the vascular lumen, accompanied by a few CD31-positive cells indicative of angiogenesis. Moreover, marked expression of integrin β1, a marker associated with cell migration [49, 56], was observed within the control vascular cavity. These findings closely aligns with the characteristic features of intimal hyperplasia seen in the control group. Differently, the TA–Cu^2+^@PLys group showed an increased proportion of PCNA-positive cells rather than integrin β1-positive cells in the vascular repair area, confirming activation of cell proliferation signaling pathways alongside inhibition of SMCs migration. Together, these findings demonstrate that the “Vascular Band-Aid” offers durable structural support, effectively modulates inflammation and accelerates cell proliferation to promote vascular regeneration, thereby suppressing post-anastomotic complications and enhancing vascular repair.

Notably, the “Vascular Band-Aid” can be speculated to exert stage-specific functions that evolve with the progression of vascular healing. During the immediate postimplantation phase (0–7 days), the “Vascular Band-Aid” provides mechanical reinforcement, ensures acute hemocompatibility through PLys surface, and mediates initial ROS scavenging by TA. In the early healing phase (3–14 days), the aligned fiber topography directs vascular cell migration, sustained Cu^2+^ release promotes angiogenesis, and the TA–Cu^2+^ reservoir continuously modulates inflammation to prevent adverse complications. During the remodeling phase (2–4 weeks and beyond), degradation of the patch facilitates gradual load transfer to the regenerating neotissue, while the bioactive interface supports maturation of the newly formed tissue. This temporal orchestration of transitioning from passive protection to active modulation exemplifies the paradigm of a proactive “Vascular Band-Aid” that operates in harmony with the natural healing cascade. Nevertheless, further optimization is warranted. For example, although the selected fiber diameter (∼1.0 μm) performs robustly in *in vitro* and *in vivo* assays, a systematic size–response study across a range of diameters, examining effects on endothelial maturation, smooth muscle phenotype and *in vivo* remodeling, would further refine the design parameters for anastomotic patch applications. Besides, the PLCL with a LA/CL = 50:50 formulation generally undergo hydrolytic degradation over 12–18 months *in vivo* [24], the 4-week *in vivo* study was therefore designed to evaluate the early-to-mid healing response to the modified patch with structural integrity maintained to support progressive tissue integration. Future long-term studies can systematically assess the degradation-remodeling dynamics over extended implantation periods.

## Conclusion

This study developed a “Vascular Band-Aid” strategy based on TA–Cu^2+^@PLys-modified aligned PLCL nanofibrous patch to proactively regulate the post-anastomotic microenvironment. This design tackles two interrelated causes of postoperative vascular failure: dysregulated host cell responses and maladaptive inflammation activation. Surface modification with TA–Cu^2+^ complexes and PLys enhances membrane wettability, surface charge and protein adsorption, collectively promoting ECs affinity and directing differentiation toward a functional mature phenotype. Concurrently, the coating confers intrinsic antioxidant activity that favorably modulates local inflammation. Applied *in situ* at the anastomotic sites, the engineered “Vascular Band-Aid” provides durable mechanical support while actively fostering vascular regeneration without adverse events. Collectively, these findings establish the efficacy of the proactive surface-engineering strategy in suppressing post-anastomotic complications, which holds particular promise for high-risk scenarios, including small-diameter vessels, postradiation or diabetic vasculature with impaired healing and patients with contraindications to systemic anticoagulation. Beyond vascular surgery, this work introduces a generalizable paradigm for biointeractive materials that integrate structural support, cell guidance and immune modulation, representing an approach that may extend to other tissue interfaces where postoperative healing failure remains a critical challenge.

## Supplementary Material

rbag164_Supplementary_Data
