# Supplementary material for: A “Vascular Band-Aid” strategy for proactive inhibition of post-anastomotic vascular complications
Source: Regen Biomater. 2026 Aug 4;13:rbag164. doi: 10.1093/rb/rbag164 (PMC13633683; doi:10.1093/rb/rbag164)
Supplement: Supplementary file 1 [file rbag164_supplementary_data.docx]

**Supporting information**

**A “Vascular Band-Aid” Strategy for Proactive Inhibition of Post-Anastomotic Vascular Complications**

# Ruijie Xu ^a, #^, Jingqiang Yan ^a, #^, Xiaojuan Li ^a, b^, Xuelian Yin ^a, b^, Zhao Yang ^a^, Qihui Zhou ^a, b,^ *, Bingcheng Yi ^a, b,^ *

**Author Affiliations:**

^a^ Department of Vascular Surgery, Qingdao Municipal Hospital, School of Life Sciences and Health, NHC Key Laboratory of Cardiopulmonary Rehabilitation and Functional Recovery, University of Health and Rehabilitation Sciences, Qingdao 266113, China.

^b^ School of Pharmacy, Qingdao University, Qingdao, 266071, China.

^#^ These authors contributed equally to this work.

*Corresponding author:

Qihui Zhou, Ph.D., Email: [qihuizhou@uhrs.edu.cn](mailto:qihuizhou@uor.edu.cn)

Bingcheng Yi, Ph.D., Email: yibingcheng@uhrs.edu.cn

**Table S1** RT-qPCR primer sequences.

| **Primer** | | **Sequence** |
| --- | --- | --- |
| *GAPDH* | *GAPDH-F* | 5’-GTCTCCTCTGACTTCAACAGCG-3’ |
|  | *GAPDH-R* | 5’-ACCACCCTGTTGCTGTAGCCAA-3’ |
| *CD31* | *CD31-F* | 5’-AAGTGGAGTCCAGCCGCATATC-3’ |
|  | *CD31-R* | 5’-ATGGAGCAGGACAGGTTCAGTC-3’ |
| *Integrin β1* | *Integrin β1-F* | 5’-CCGAAGAGGTACTTGTTGCAGC-3’ |
|  | *Integrin β1-R* | 5’-GGCTTCCGTGAATGCCTCCTTT-3’ |
| *MLCK* | *MLCK-F* | 5’-GAGGTGCTTCAGAATGAGGACG-3’ |
|  | *MLCK-R* | 5’-GCATCAGTGACACCTGGCAACT-3’ |
| *VE-cadherin* | *VE-cadherin-F* | 5’-GAAGCCTCTGATTGGCACAGTG-3’ |
|  | *VE-cadherin-R* | 5’-TTTTGTGACTCGGAAGAACTGGC-3’ |

**Figure S1** The average fibre diameter of the "Vascular Band-Aid": (**A-C)** Fiber diameter distribution of PLCL, TA-Cu^2+^ and TA-Cu^2+^@PLys, respectively; (**D)** Comparison in fiber diameter. Stepwise modification with TA-Cu^2+^ complexes and PLys molecules progressively increased the average fibre diameter, from 1.01 ± 0.16 μm for pristine PLCL to 1.07 ± 0.12 μm for TA-Cu^2+^ and 1.15 ± 0.22 μm for TA-Cu^2+^@PLys.

**Figure S2** The surface roughness of single fiber in the "Vascular Band-Aid". Stepwise modification with TA-Cu^2+^ complexes and PLys molecules progressively increased the fibre surface roughness, from 3.64 ± 1.71 nm for pristine PLCL to 11.39 ± 3.69 nm for TA-Cu^2+^ and 13.37 ± 1.03 nm for TA-Cu^2+^@PLys.

**Figure S3** Surface chemical characterizations of the "Vascular Band-Aid": (**A)** FTIR spectra; (**B)** XPS spectra of TA-Cu^2+^@PLys for the deconvolution of N1s peaks. The N1s core level spectrum can be curve-ﬁted with three peaks at 395.0 eV (−N=), 399.6 eV (−NH−), and 401.2 eV (−NH_2_).

**Table S2** Surface functional group composition calculated from XPS spectra.

**Figure S4** The kinetic clotting time of the “Vascular Band-Aid” for 50 min. Compared with PLCL, TA-Cu^2+^@PLys tended to induce less clot formation.

**Figure S5** Antioxidant capacity of the “Vascular Band-Aid”. TA-Cu^2+^@PLys membrane exhibits significant free radical scavenging activity, with DPPH and ABTS radical scavenging rates reaching 76.34% and 86.96%, respectively, which are substantially higher than those of unmodified PLCL.

**Figure S6** The quantified TGF-β/TNF-α ratio from immunohistochemical staining images of samples at 4 W. Compared to control, TA-Cu^2+^@PLys significantly increased the TGF-β/TNF-α ratio at the vascular anastomotic sites, indicating a shift towards an anti-inflammatory state.
